# An Above-Average Lymph Node Yield Removed During Curative Neck Dissection in Advanced Head and Neck Squamous Cell Carcinomas Improves Survival

**DOI:** 10.3390/cancers18010068

**Published:** 2025-12-25

**Authors:** Miray-Su Yılmaz Topçuoğlu, Thiemo Seidler, Patrick J. Schuler, Christel Herold-Mende, Rolf Warta, Gerhard Dyckhoff

**Affiliations:** 1 Department of Otorhinolaryngology, Head and Neck Surgery, Medical Faculty of Heidelberg, University Hospital Heidelberg, 69120 Heidelberg, Germany; thiemo.seidler@icloud.com (T.S.); patrick.schuler@med.uni-heidelberg.de (P.J.S.); christel.herold-mende@med.uni-heidelberg.de (C.H.-M.); rolf.warta@med.uni-heidelberg.de (R.W.); gerhard.dyckhoff@med.uni-heidelberg.de (G.D.); 2 Division of Experimental Neurosurgery, Department of Neurosurgery, Medical Faculty of Heidelberg, University Hospital Heidelberg, 69120 Heidelberg, Germany

**Keywords:** HNSCC, lymph node yield, neck dissection, outcome, survival

## Abstract

**Objectives:** The lymph node yield of a curative neck dissection for advanced head and neck squamous cell carcinoma (HNSCC) is an important factor in improving patient outcomes. Achieving adequate resection while minimising surgical risk is important. This retrospective study investigated the role of the lymph node yield for the survival of patients with HNSCC. **Methods:** A total of 234 patients with advanced HNSCC who were treated at a German university hospital between 1997 and 2018 were analysed. The analysis included patient data, tumour-specific characteristics, and the extent of neck dissection performed. Statistical analysis was performed using multivariate Cox proportional hazards models, supplemented by Kaplan–Meier analyses. **Results:** The median age was 60.0 years (range: 30–85 years, interquartile range: 12.3 years). The follow-up period covered up to 25 years. According to the Union for International Cancer Control, *n* = 64 patients had UICC-stage III, and 170 patients had UICC-stage IV. The above-average lymph node yield was superior to average, but especially to below-average lymph node yields in bilateral (2.9-fold), ipsilateral (2.6-fold), and contralateral (10.7-fold) neck dissection. In particular, contralateral neck dissection was found to correlate with a significantly better prognosis in terms of overall survival when a higher number of lymph nodes were removed. **Conclusions:** The study suggests that a thorough and careful neck dissection involving the removal of a greater number of lymph nodes and including the contralateral side could significantly improve the survival for patients with advanced HNSCC.

## Simple Summary

For appropriate treatment, lymph nodes must be removed in advanced squamous cell cancers of the head and neck in a procedure called neck dissection. The surgeon’s aim is to minimise surgical risk while achieving the best possible outcome, so the question of how many lymph nodes should be resected in these cases to gain a long survival remains a topic of discussion. The patient data, surgery information, and outcome of a total of 234 patients with this type of cancer who were treated at a German university hospital between 1997 and 2018 were investigated. The study showed that a thorough neck dissection involving an above-average number of nodes can improve survival rates, especially when performed on the side opposite the primary tumour.

## 1. Introduction

Head and neck cancers are the sixth most common type of tumours worldwide [1,2,3,4] and are currently showing a growing incidence in younger patients due to the increased association with human papilloma virus (HPV) infection [5,6]. Histologically, head and neck cancers are predominantly squamous cell carcinomas (HNSCC) [7,8]. The 5-year survival rate ranges between 30% and 80%, depending on the initial tumour stage, the presence of lymph nodes and/or distant metastases, the p-16 status, and the occurrence of recurrence [1,9,10,11,12,13]. Depending on the location and tumour stage, prevalent metastases in lymph nodes or distant locations, the general condition of the patient, and the risk factors present, a choice is made between the available treatment modalities [13,14,15].

In consideration of the prevalence of occult lymph node metastases in HNSCC with 12–27% despite the availability of advanced imaging modalities [16,17,18,19,20], and the significant meaning of lymph node metastases for survival outcomes (N0 67.9%, N+ 39.9%) [16], the question arises as to the optimal count of lymph nodes to be resected in order to reduce the likelihood of occult metastases in situ, and consequently to reduce recurrence risk and enhance survival rates [21,22,23,24,25].

It is known that an increased lymph node yield increases the likelihood of finding cervical lymph node metastases [21,26]. Ebrahimi et al. presented a minimum of 18 lymph nodes as sufficient nodal yield after neck dissection in oral squamous cell cancer to correctly describe the nodal stage and improve survival [23,24]. Based on these studies of Ebrahimi et al., Divi et al. also chose 18 lymph nodes as the cut-off number of lymph nodes to investigate the oncologic outcome of patients with mucosal SCC [27]. Divi et al. also could show improved survival and lower rates of local tumour recurrences for node yields over 18 lymph nodes [27]. Also, Merz et al. found that a lymph node yield of at least 15 was significant for improved survival [28]. They also investigated the impact of a higher nodal yield of over 40 lymph nodes in node-negative patients [28]. It was shown that survival was better with more than 15 resected lymph nodes [28]. A total of 80% of their study group consisted of patients with T1/2 HNSCC [28]. By contrast, Ryu et al. resected a median of 52 lymph nodes, but focused more on the role of positive lymph nodes and lymph node density in survival than on identifying an optimal number of lymph nodes to be resected [29].

Zhuge et al. could show, that for T1/T2 supraglottic laryngeal squamous cell cancer, the survival improves and the mortality decreases in patients who underwent neck dissection with more than ten lymph nodes extirpated [22]. Also, they stated that an insufficient number of resected lymph nodes in neck dissection can cause false-negative nodal staging [22].

Focussing on oral tongue squamous cell carcinoma, Zhuge et al. found a count of 15 lymph nodes to be optimal for the postoperative prognosis [21]. They also showed that a lymph node yield of more than 19 nodes increased the probability of finding metastatic lymph nodes [21]. Agrama et al. found more than 20 lymph nodes sufficient to increase the probability of finding lymph node metastases [26].

Still, a comprehensive overview of the entities of HNSCC is missing, while Zhuge et al., Lemieux et al., and Ebrahimi et al. focussed on subspecialities of tumours, such as supraglottic larynx carcinoma and oral carcinoma [22,23,24,25], Agrama et al. and Divi et al. focussed on the oral cavity, oropharynx, and hypopharynx, but only focussed on the likelihood of finding occult lymph node metastases and not on the survival depending on the count of harvested lymph nodes [26], and pre-determined their cut-off to be 18 lymph nodes [27]. Merz et al. additionally investigated the impact of resecting more than 40 lymph nodes on the survival of patients with HNSCC and node-negative neck [28]. However, they presented a study cohort with less-advanced tumours [28]. In short, previous studies have identified a cut-off value of 15–20 lymph nodes as sufficient for a successful yield. To the best of our knowledge, data on the issue of whether harvesting even more lymph nodes could further improve patients’ outcomes are scarce.

The aim of this study was therefore to investigate the impact of a more broadly chosen range of resected lymph nodes during neck dissection on the survival of patients with HNSCC, without predetermining a cut-off point for the number of lymph nodes. The current study showed that an above-average yield of lymph nodes improves survival.

## 2. Materials and Methods

### 2.1. Patients

A single-centred, retrospective data analysis was performed on a total of 234 patients with advanced HNSCC. The study was approved by the local ethics committee (protocol S-70/99, amendment 9 January 2004), and written informed consent was obtained from all included patients. All patients were treated and followed up with for up to 25 years at the Department of Otorhinolaryngology, Head and Neck Surgery at a German university hospital between March 1997 and May 2018. The cohort included patients with stage III and IVa/b tumours (determined as per Union for International Cancer Control (UICC)) who underwent primary curative resection of the primary cancer and therapeutic neck dissection and risk-adapted adjuvant radio(chemo)therapy. Further inclusion criteria were the proven histopathology of HNSCC, available detailed histopathological results with exact count and side of the resected lymph nodes, documented size of the lymph nodes, and the localisation of the tumour in the oral cavity, oropharynx, hypopharynx, or larynx. Relevant data were retrieved from the digital patient records (Table 1). The patients’ overall survival was assessed.

The number and location of the resected lymph nodes were obtained from archived pathology reports. To evaluate the prognosis depending on the number of lymph nodes removed, the cases were divided into three groups. In ipsilateral and contralateral neck dissection, 10–30 removed lymph nodes were considered to be the usual average, based on the earlier literature [21,22,23,24,27,30,31,32], with numbers below (<10 lymph nodes) and above (>30 lymph nodes) this range defined as below- and above-average groups, respectively. For bilateral neck dissection, the average was adjusted to 20–50 lymph nodes removed, with numbers below (<20 lymph nodes) and above (>50 lymph nodes) this range defined as below- and above-average groups, respectively. The addition of a third ‘above-average’ group allowed us to examine the impact of removing a greater number of lymph nodes on prognosis. The number to define the above-average group in bilateral neck dissection was not simply doubled from the ipsilateral neck dissection, as this would no longer reflect actual clinical practice. Only 4.4% of cases involved resection of more than 30 lymph nodes on the contralateral side, so assuming an above-average total number of more than 60 lymph nodes in bilateral neck dissection would have been unreasonable.

### 2.2. Statistical Analysis

The descriptive data and figures were analysed and computed using GraphPad Prism version 10.4.0 (GraphPad Software, Boston, MA, USA). Descriptive data were presented as the median, range, and interquartile range (IQR). For the investigation of the survival data and the multivariate analysis, the programme RStudio was used (Posit team (2025). RStudio: Integrated Development Environment for R version 2025.009.0+387, Boston, MA, USA). The likelihood ratio test was performed to check whether covariates’ age, gender, cancer localisation, extracapsular spread, T-/N-classification, UICC stage, treatment, and count of resected lymph nodes were likely to influence survival rates. Covariates that were significant in the likelihood ratio tests (value ≤ 0.1) were then included in a multivariate Cox proportional hazards model (CoxPH-model) and were kept constant for the computing of survival data. Kaplan–Meier curves were created to visualise the results, showing the isolated influence of the number of lymph nodes removed on survival with relevant covariates kept constantly. Also, a direct pairwise log-rank test to compare the survival within all three groups was performed, calculating the *p*-values. Also, the hazard ratio (HR), and the 95%-confidence interval (95%-CI), based on the CoxPH-model were calculated. In each pairwise comparison, the group with the lower number of resected lymph nodes served as the reference group. The Benjamini–Hochberg adjustment was performed to control for errors associated with multiple testing. The significance level was set to *p* < 0.05.

For testing, a distinction was made between unilateral and bilateral neck dissection, as this also correlates with the lymph node yield and, consequently, the thoroughness of the neck dissection. The different groups were

1. Count of resected lymph nodes during bilateral neck dissection.
2. Count of ipsilaterally resected lymph nodes during ipsilateral neck dissection.
3. Count of contralaterally resected lymph nodes during bilateral neck dissection.

## 3. Results

### 3.1. Patient Data and Cancer Characteristics

**Figure 1:**
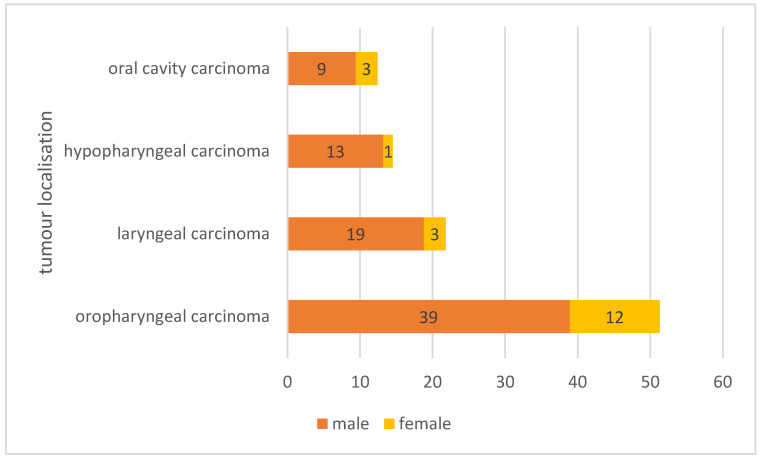
Relative distribution of the study cohort’s cancer localisations (*n* = 234) subdivided by gender distribution in percentage numbers.

**Figure 2:**
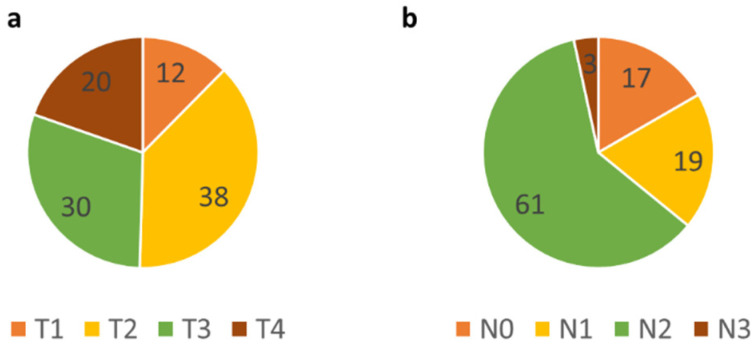
Relative distribution of T-classification (**a**) and N-classification (**b**) in the patient cohort of *n* = 234 patients.

In total, 234 patients were included. The median age at diagnosis was 60.0 years (range: 30–85 years, IQR: 12.3). The follow-up period covered up to 25 years. The patient cohort consisted of 188 male (80.3%), and 46 female (19.7%) patients (Figure 1, Table 2). Of the included patients, 86 patients were smokers (36.8%), and 76 patients consumed alcohol regularly (32.5%). Oropharyngeal carcinomas (*n* = 120; 51.3%) were the most common cancers in the study cohort, followed by laryngeal carcinomas (*n* = 51; 21.8%), hypopharyngeal carcinomas (*n* = 34; 14.5%), and oral cavity carcinomas (*n* = 29; 12.4%) (Figure 1). Details on cancer characteristics and performed adjuvant treatment modalities are presented in Figure 2 and Table 2, respectively.

A bilateral neck dissection was performed in *n* = 158 patients (67.5%), while a sole ipsilateral neck dissection was performed in *n* = 76 patients (32.5%). Pathological examination revealed extracapsular spread in *n* = 116 patients (49.6%).

### 3.2. Survival

#### 3.2.1. A Higher Total Count of Resected Lymph Nodes in Bilateral Neck Dissection Significantly Improves Survival

At the 158 bilateral neck dissections, in median *n* = 36.5 lymph nodes were resected (range: 5–82; IQR: 22.3). A total of 97 patients in this group died. Of these, 38 (39%) died from tumour-related causes, while 59 (61%) patients died from other causes. In *n* = 37 patients (23.4%), more than 50 lymph nodes were resected, in *n* = 104 patients (65.8%), 20 to 50 lymph nodes were resected, and in *n* = 17 patients (10.8%), fewer than 20 lymph nodes were resected.

**Figure 3:**
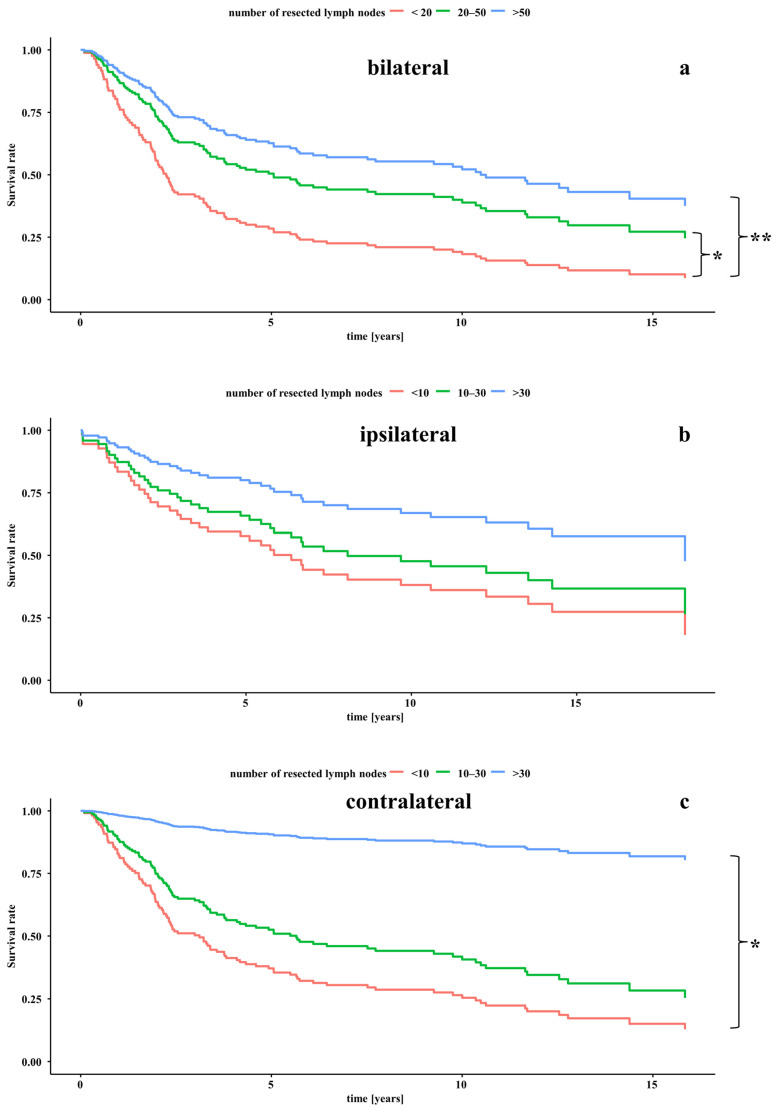
Kaplan–Meier curves of the survival depending on the count of removed lymph nodes of (**a**) bilateral neck dissections, (**b**) ipsilateral neck dissections, and (**c**) contralateral neck dissections. Survival rates over 15 years depending on bilateral (**a**), ipsilateral (**b**), and contralateral (**c**) lymph node yields demonstrated with the Kaplan–Meier curves. The relevant covariates were kept constant. * significant difference in survival between groups with a significance level of *p* <0.05. ** significant difference in survival between groups with a significance level of *p* < 0.01.

The calculation of the likelihood ratio revealed the extracapsular spread, the N-classification, and the UICC stage as significant covariates for survival (Table 3). Thus, these covariates were held constant in the multivariate CoxPH analysis for the bilateral neck dissections for the computing of the Kaplan–Meier curves to demonstrate the sole impact of the count of resected lymph nodes on the patients’ survival data (Figure 3a). When compared to the below-average group there was significantly improved survival in the groups with a yield of 20–50 lymph nodes (HR 1.93; 95%-CI 0.28–0.96; *p* < 0.05), and a yield of >50 lymph nodes (HR 2.88; 95%-CI 0.16–0.73; *p* < 0.01) (Table 4). When comparing the survival of the above-average group with >50 lymph nodes resected to those with a yield of 20–50 lymph nodes, there was a trend towards a 1.49-fold improved survival in the above-average group, although without statistical significance (HR 1.49; 95%-CI 0.40–1.14; *p* = 0.139) (Table 4).

#### 3.2.2. A Higher Count of Resected Lymph Nodes in Ipsilateral Neck Dissection Tends to Improve Survival

At the 76 solely ipsilateral neck dissections, in median, *n* = 21.5 lymph nodes were resected on the ipsilateral side (range: 3–67; IQR: 12.0). A total of 36 patients in this group died. Of these, 12 (33%) died from tumour-related causes, while 24 (67%) patients died from other causes. In *n* = 15 patients (19.7%) >30 lymph nodes were resected ipsilaterally, in *n* = 54 patients (71.1%) 10 to 30 lymph nodes were resected, and in *n* = 7 patients (9.2%) fewer than 10 lymph nodes were resected.

The calculation of the likelihood ratio revealed the localisation and the extracapsular spread as significant covariates for survival (Table 3). Thus, these covariates were held constant in the multivariate CoxPH analysis for the ipsilateral neck dissections for the computing of the Kaplan–Meier curves to demonstrate the sole impact of the count of resected lymph nodes on the patients’ survival data (Figure 3b). When comparing to the below-average group with fewer than 10 lymph nodes resected, there was only a trend towards improved survival in the groups with a yield of 10–30 lymph nodes (HR 1.34; 95%-CI 0.24–2.80; *p* = 0.605), and a yield of >30 lymph nodes (HR 2.62; 95%-CI 0.09–1.59; *p* = 0.187) (Table 4). Also, when comparing the survival between the patients with a yield of >30 lymph nodes to those with a yield of 10–30 lymph nodes, there was a trend towards a 1.95-fold improved survival in the above-average group (HR 1.95; 95%-CI 0.17–1.52; *p* = 0.230) (Table 4).

#### 3.2.3. A Higher Count of Contralaterally Resected Lymph Nodes in Bilateral Neck Dissection Significantly Improves Survival

In cases where bilateral neck dissection was performed (*n* = 158), on the contralateral side in median *n* = 15.0 lymph nodes were resected (range: 1–43; IQR: 10.3). A total of 97 patients in this group died. Of these, 38 (39%) died from tumour-related causes, while 59 (61%) patients died from other causes. Contralaterally, in *n* = 7 patients (4.4%) >30 lymph nodes were resected, in *n* = 119 patients (75.3%) 10 to 30 lymph nodes were resected, and in *n* = 32 patients (20.3%) fewer than 10 lymph nodes were resected.

The calculation of the likelihood ratio revealed the extracapsular spread, the N-classification, the UICC stage, and the count of removed lymph nodes as significant covariates for survival (Table 3). Thus, these covariates were held constant in the multivariate CoxPH analysis for the contralateral neck dissections for the computing of the Kaplan–Meier curves to demonstrate the sole impact of the count of resected lymph nodes on the patients’ survival data (Figure 3c). When comparing to the group that received a neck dissection with a yield of <10 lymph nodes, there was a 1.58-fold trend towards an improved survival in the average group with a yield of 10–30 lymph nodes (HR 1.58; 95%-CI 0.39–1.04; *p* = 0.669), and a statistically significantly improved survival rate in the above-average group with a yield of >30 lymph nodes (HR 10.68; 95%-CI 0.01–0.70; *p* = 0.021) (Table 4). Also, when comparing the above-average group with the average group, there was a trend to a 6.77-fold improved survival in the above-average group (HR 6.77; 95%-CI 0.02–1.07; *p* = 0.058) (Table 4).

## 4. Discussion

Frequent and early lymph node metastases, as well as occult metastases, complicate the treatment of HNSCC, and prevalent lymph node metastases increase the risk of recurrences and decrease disease-specific survival [10,15,16,17,19,20,33,34]. The nodal stage is relevant for decision making on treatment paths, and, thus, its assessment needs to be precise [21,35]. The impact of neck dissections on survival has already been investigated for many different tumour types, and it is a current topic of research as to what count of lymph nodes should be resected to optimise survival outcome [35,36].

Our study investigated the impact of the lymph node yield in neck dissections in advanced HNSCC. It could be shown that a thorough resection of lymph nodes in neck dissections has a significant positive effect on survival.

The patient cohort presented mirrors the epidemiologic distribution, with male patients being affected more frequently than females [37], and the median age at diagnosis being 60 years [38].

The study showed that, while there was a positive trend towards improved survival with an increasing number of lymph nodes removed for ipsilateral neck dissection, this was particularly significant for contralateral and bilateral neck dissection.

In the literature, there has been much discussion about which cut-off values should be chosen for a sufficient neck dissection [30,31]. The absolute minimal nodal yield was reported to be ten to still reach a relevant impact on patients’ survival [30,31], and higher nodal yields has been a quality indicator for neck dissections [30,39].

Ebrahimi et al. categorised the lymph node yield into four groups: <18, 18–24, 24–32, and >32 lymph nodes in oral cavity carcinomas with low tumour stages [24]. Ebrahimi’s analysis revealed that the prognosis improves significantly with the removal of 18 or more lymph nodes, but no further significant improvement was observed with the removal of more than 18, 24 or 32 nodes [23,24]. Therefore, the plateau reached with a lymph node yield of 18 could be due to the more favourable overall prognosis of the early stages examined in Ebrahimi’s study, in which additional surgical gain might be of limited meaning for prognosis [24], and the number of resected lymph nodes holds a more diagnostic value in terms of detecting occult lymph node metastases. Other authors have also defined the number of around 18 lymph nodes that should be minimally resected in order to improve survival rates [27,28,39,40,41]. This has become standard practice. However, the significance of an above-average number of resected lymph nodes has not been explicitly investigated in these studies, and is a new aspect of our study, especially in advanced tumours. Iocca et al. found a cut-off value for adequate lymph node yields in curative neck dissections of HNSCC of around 16 lymph nodes as too low [31], given the fact that, on average, 34 lymph nodes are resected in neck dissections of the oral cavity and oropharyngeal carcinomas [32].

Our analysis of all types of neck dissection (bilateral, ipsilateral, and contralateral) has consistently shown that the average and above-average groups have a survival advantage over the below-average group. Consequently, our data show that survival is improved by an increased number of resected lymph nodes. In particular, our data suggest that removing a sufficiently high number of lymph nodes during bilateral and contralateral neck dissection appears to positively impact survival rates. This may be because a thorough bilateral and contralateral neck dissection increases the likelihood of detecting occult lymph node metastases. The rate of occult lymph nodes will be reported in our future work. Additionally, the detection of occult lymph node metastases leads to a more accurate classification of the extent of the tumour, enabling more precise therapy planning and reducing the risk of recurrence, next to an improved survival. Taken together, the study suggests that more extensive neck dissections may benefit patients with advanced tumours, and that the number of lymph nodes resected is a valuable indicator of quality. The authors recommend resection of at least 30 lymph nodes for ipsilateral neck dissection and at least 50 for bilateral neck dissection, particularly for treatment-naïve patients with advanced HNSCCs.

Still, while a more extensive neck dissection with a greater yield of lymph nodes has a positive impact on survival rates and specifies staging and decision making, the postoperative quality of life of patients should be preserved as much as possible. Any reversible and irreversible impairments caused by neck dissection, such as nerve damage, lymphoedema, swallowing disorders, restricted mobility, and pain should be minimised [42,43,44]. This means that the surgeon must strike a balance between adverse surgical consequences, because of the most thorough neck dissection feasible and maintaining life quality as best as possible.

This study had several limitations. Firstly, only advanced HNSCC in UICC stage III, IVa/b were included to obtain a homogeneous group of advanced tumours for which therapeutic neck dissection was definitely indicated. As our study consequently only included patients who underwent therapeutic neck dissections, our results do not apply to cT1/2 cN0 patients who underwent elective neck dissection for particularly diagnostic purposes. Thus, on the base of this study, the role of the count of lymph node yield in neck dissections cannot be evaluated for UICC stages I and II. Secondly, we carried out a monocentre study, with an underrepresentation of patients with tumours of the oral cavity. In our university hospital, patients of this tumour site receive mostly surgery in the department of oral and maxillofacial surgery. This study included a relatively small number of patients (*n* = 234), which should be increased to verify the study findings and to be more representative. A larger sample size would also strengthen the subgroup analysis based on the number of lymph nodes resected. Thus, the results and conclusions that are drawn from this study only apply to the study cohort and cannot be generalised for all patients with HSNCCs. Additionally, the survival analysis did not differentiate between various tumour locations, which impact survival due to the differences in lymphatic drainage and treatment strategies. An analysis of specific tumour localisation in relation to nodal status has yet to be performed with an equally distributed study cohort in relation to the tumour localisation and an adapted and adequate cohort size to perform subgroup analysis. In the future, a multicentre study could provide more insights into this topic. Another aspect is, that in the context of HNSCC, a relevant and topical issue is HPV-associated oropharyngeal carcinomas. These tumours differ from non-HPV HNSCCs in terms of histology, spread, treatment approaches, and prognosis. As awareness of this only emerged in the 2010s, these tumours were not considered separately in our evaluations, as our data analysis began in 1997. Next, the number of lymph nodes resected depends not only on the surgeon, but also on the pathologists’ processing procedure and experience [30]. The fact that the study period spans two decades is on the one side a major strength of the study, but at the same time also a limitation as it additionally introduces variability regarding diagnosis, staging systems, and treatment approaches. Also, the attending pathologists and surgeons partly changed over the course of the twenty years. Further, diagnostic methods for detecting small lymph node metastases were certainly not as effective at the end of the 1990s as they are today with thin-layer computed tomography. This may have influenced the frequency of actual performed ipsi- and bilateral neck dissections. Additionally, what would have been defined as occult lymph node metastasis after histological confirmation at that time, because it was not diagnosed earlier in imaging, might be detected prior to surgery today. This could influence the surgeon’s approach (less vs. more extensive). In turn, this could have affected the number of lymph nodes resected and thus the main investigational objects of our study. In the meantime, significant advances have been made in radio- and chemotherapeutic tumour therapies in recent years, meaning that some patients in the original cohort would probably not have undergone surgery, particularly those with positive p16 status. Furthermore, as p16-positive patients are known to have a better survival rate, the missing p16 status is an unknown confounder of the survival analysis of the current study. Unfortunately, this limitation could not be easily overcome because the p16 status was not documented until the 2010s, as already discussed above. The same applies for accompanying survival-affecting comorbidities, which could not easily be achieved from patients from the former decades. Altogether, these variabilities are a natural limitation of long-term studies, which we accepted in order to report on the still valuable long-term observations.

On the other side, this study had several strengths. Unlike previous studies, this study examined not only the outcome of a below- and above-average nodal yield of a fixed cut-off value for resected lymph nodes, but also the significance of an above-average nodal yield after neck dissection. Another strength of the study was the performance of a multivariate analysis, which took several influencing factors into account simultaneously in order to eliminate the effect of any confounding factors. The CoxPH-model is established for reliable risk analyses. A further strength was that this study evaluated the survival period up to 25 years, which is longer than that of other comparable studies, which range from 5 to 10 years [21,22,23,24]. This allows for a longer observation period. Therefore, differences that were not yet relevant after a 5-year observation period could become relevant after a 15-year observation period.

In the future, improvements in the accuracy of imaging techniques, such as high-resolution magnetic resonance imaging with deep learning technologies and other technological advances, are expected to enable a more accurate determination of the number and distribution of cervical lymph nodes, as well as discovering occult and non-occult lymph node metastases [33].

## 5. Conclusions

In conclusion, the treatment aim should be to perform a thorough neck dissection without increasing the radicality of surgery and without reducing the quality of life by causing postoperative local impairments. Our study showed that a lymph node yield of over 30 lymph nodes in contralateral neck dissection, and of over 50 lymph nodes in bilateral neck dissection, improves survival in the investigated study cohort.

As precise identification of positive lymph node metastases is essential for optimal diagnosis and therapy planning, and as thorough neck dissection increases survival rates, the removal of as many lymph nodes as possible per resected lymph node level should be performed in a risk–benefit evaluation as part of curative therapy for advanced HNSCCs, also from the contralateral side. To further verify the results of this study, the study design needs to be expanded in the future. This will require the study groups to be further subdivided according to tumour location, elective versus therapeutic neck dissection or p16 status, and a larger cohort, preferably multicentre, to be examined.

## Figures and Tables

**Table 1:** Data retrieved from digital patient records.

Patient data	Age at diagnosis	
	Gender	
	Risk factors	
	Comorbidities	
Treatment data	Surgical approach and technique	
	Side and type of performed neck dissection	
	Adjuvant treatment	
Histopathology	*Tumour*	*Lymph nodes*
	Localisation	Side
	Size	Count of
	Grading	-resected lymph nodes per side
	R-stage	-N+ lymph nodes
		-maximum diameter of
		biggest lymph node metastasis
		-extracapsular spread
TNM-stage UICC-stage		
Oncological follow-up	Local recurrences	
	Nodal recurrences	
	Secondary HNSCC cancer	
	Late metastases	
Survival Data	Date of death Cause for death Progress-free survival Recurrence-free survival Survival interval free of a secondary cancer Interval free of late metastases Overall survival	

R-stage: Presence of tumour tissue after resection; N+: prevalent lymph node metastases; UICC: Union for International Cancer Control; HNSCC: head and neck squamous cell cancer.

**Table 2:** Demographics, cancer characteristics, and adjuvant treatment.

Feature	Category	Cases in Total *n* = 234	Cases Bilateral *n* = 158	Cases Ipsilateral *n* = 76
Age	<60	117 (50)	80 (51)	37 (49)
	≥60	117 (50)	78 (49)	39 (51)
Gender	Male	188 (80)	132 (84)	56 (74)
	Female	46 (20)	26 (16)	20 (26)
Localisation	Oral cavity	29 (12)	22 (14)	7 (9)
	Oropharynx	120 (51)	60 (38)	60 (79)
	Larynx	51 (22)	48 (30)	3 (4)
	Hypopharynx	34 (15)	28 (18)	6 (8)
Extracapsular spread	Negative	118 (50)	80 (51)	38 (50)
	Positive	116 (50)	78 (49)	38 (50)
T-classification	T1	29 (12)	16 (10)	13 (17)
	T2	89 (38)	51 (32)	38 (50)
	T3	70 (30)	54 (34)	16 (21)
	T4	46 (20)	37 (23)	9 (12)
N-classification	N0	39 (17)	34 (22)	5 (7)
	N1	45 (19)	26 (16)	19 (25)
	N2	142 (61)	92 (58)	50 (66)
	N3	8 (3)	6 (4)	2 (3)
UICC stage	III	64 (27)	41 (26)	23 (30)
	IV	170 (73)	117 (74)	53 (70)
Adjuvant treatment	None	46 (20)	32 (20)	14 (18)
	RTx	91 (39)	59 (37)	32 (42)
	RCTx	97 (41)	67 (42)	30 (39)

In total 234 patients could be included. *n* = 158 received bilateral; neck dissection; *n* = 76 received ipsilateral neck dissection; data given as absolute numbers n (relative numbers in %); UICC: Union for International Cancer Control; only UICC stage III, IVa, and IVb were included; RTx: radiation only; RCTx: radiochemotherapy.

**Table 3:** Likelihood ratios.

Neck Dissection	Bilateral	Ipsilateral	Contralateral
Age	0.9	0.2	0.9
Gender	0.7	0.5	0.7
Localisation	0.6	**0.1**	0.6
Extracapsular spread	**0.04**	**0.04**	**0.04**
T-classification	0.7	0.3	0.7
N-classification	**0.04**	0.6	**0.04**
UICC stage	**0.04**	0.6	**0.04**
Adjuvant treatment	0.2	0.3	0.2
Count of removed Lymph nodes	0.3	0.3	**0.01**

Ratios of ≤0.1 were defined as significant covariates and marked in bold.

**Table 4:** Inter-group comparison of survival data after neck dissections (ND) with log-rank test.

ND-Groups	<20 vs. 20–50		<20 vs. >50		20–50 vs. >50	
	**HR (95%-CI)**	***p*** **-value**	**HR (95%-CI)**	***p*** **-value**	**HR (95%-CI)**	***p*** **-value**
Bilateral	**1.93** (0.28–0.96)	**0.036**	2.88 (0.16–0.73)	**0.005**	1.49 (0.40–1.14)	0.139
	**<10 vs. 10–30**		**<10 vs. >30**		**10–30 vs. >30**	
	**HR (95%-CI)**	***p*** **-value**	**HR (95%-CI)**	***p*** **-value**	**HR (95%-CI)**	***p*** **-value**
Ipsilateral	1.34 (0.24–2.28)	0.605	2.62 (0.09–1.59)	0.187	1.95 (0.17–1.52)	0.230
Contralateral	1.58 (0.39–1.04)	0.069	10.68 (0.01–0.70)	**0.021**	6.77 (0.02–1.07)	0.058

HR (95%-CI): hazard ratio (95% confidence intervals lower-upper border) and *p*-value of survival data comparing groups with each other. Bold: *p*-values < 0.05. HR: hazard ratio; CI: confidence interval. The headings are highlighted in grey.

## Data Availability

The original contributions presented in this study are included in the article. Further inquiries can be directed to the corresponding author.
